# The Major Hypotheses of Alzheimer’s Disease: Related Nanotechnology-Based Approaches for Its Diagnosis and Treatment

**DOI:** 10.3390/cells12232669

**Published:** 2023-11-21

**Authors:** César Cáceres, Bernardita Heusser, Alexandra Garnham, Ewa Moczko

**Affiliations:** Facultad de Ingeniería y Ciencias, Universidad Adolfo Ibáñez, Viña del Mar 2562307, Chile; cesar.caceres@uai.cl (C.C.);

**Keywords:** neurodegeneration, neuroscience, Alzheimer’s disease, nanotechnology, biomarkers, drug delivery, nanoparticles

## Abstract

Alzheimer’s disease (AD) is a well-known chronic neurodegenerative disorder that leads to the progressive death of brain cells, resulting in memory loss and the loss of other critical body functions. In March 2019, one of the major pharmaceutical companies and its partners announced that currently, there is no drug to cure AD, and all clinical trials of the new ones have been cancelled, leaving many people without hope. However, despite the clear message and startling reality, the research continued. Finally, in the last two years, the Food and Drug Administration (FDA) approved the first-ever medications to treat Alzheimer’s, aducanumab and lecanemab. Despite researchers’ support of this decision, there are serious concerns about their effectiveness and safety. The validation of aducanumab by the Centers for Medicare and Medicaid Services is still pending, and lecanemab was authorized without considering data from the phase III trials. Furthermore, numerous reports suggest that patients have died when undergoing extended treatment. While there is evidence that aducanumab and lecanemab may provide some relief to those suffering from AD, their impact remains a topic of ongoing research and debate within the medical community. The fact is that even though there are considerable efforts regarding pharmacological treatment, no definitive cure for AD has been found yet. Nevertheless, it is strongly believed that modern nanotechnology holds promising solutions and effective clinical strategies for the development of diagnostic tools and treatments for AD. This review summarizes the major hallmarks of AD, its etiological mechanisms, and challenges. It explores existing diagnostic and therapeutic methods and the potential of nanotechnology-based approaches for recognizing and monitoring patients at risk of irreversible neuronal degeneration. Overall, it provides a broad overview for those interested in the evolving areas of clinical neuroscience, AD, and related nanotechnology. With further research and development, nanotechnology-based approaches may offer new solutions and hope for millions of people affected by this devastating disease.

## 1. Introduction

Neurodegenerative diseases are age-related pathologic conditions, which, similar to other chronic diseases, such as cancers or cardiovascular diseases, are common, long-lasting, progressive health disorders [1,2]. They are the main cause of senile dementias, which progress with cognitive deterioration, loss of daily independence, and, at the cellular level, synaptic failure. They start quietly and gradually advance over the years. Why is research on those diseases so important? Even if possible risk factors for some of them have been identified, the origins still remain unknown. Generally, chronic conditions cannot be prevented by vaccination or easily cured by medication. Therefore, scientists are forced to use their knowledge and skills and combine disciplines to propose more efficient and cost-effective ways to take steps forward.

Alzheimer’s disease (AD) is the most prevalent form of dementia, and it affects millions of people worldwide, especially aged people [3,4,5,6]. This neurodegenerative disorder leads to the progressive death of neurons and, inevitably, memory loss, the ability to communicate coherently and learn new information, and the progressive need for full-time care. In March 2019, one of the major pharmaceutical companies and pioneers in neuroscience announced that it cancelled all clinical trials of new drugs, and there is currently no cure for Alzheimer´s. The reason for this was the high cost and lack of sufficient evidence supporting the efficiency of the treatments. It sounded horrifying because watching a friend or family member struggle with AD can be heartbreaking. In June 2021, the Food and Drug Administration (FDA) approved the first drug to treat Alzheimer’s, aducanumab. The approval was based on the data that patients taking aducanumab demonstrated improvements compared to those taking a placebo in cognitive tests. The uncertainty of this information and the clinical benefits of the drug were questioned by the Centers for Medicare and Medicaid Services. As a result, they decided not to cover the high cost of the treatment and required additional trials to confirm the effectiveness of the therapy. This again left many people without hope [7]. Recently, in January 2023, the FDA accepted the second-ever drug for AD, lecanemab. The decision was again very controversial. It has been proven that lecanemab slows down the progression of the disease in its early stages, but the data that were taken into account included only phase I and phase II of the clinical trials. Researchers are also seriously concerned about the reports of the deaths of patients exposed to the longer treatment. Therefore, the world still suffers from the lack of a clear cure for AD.

**Figure 1:**
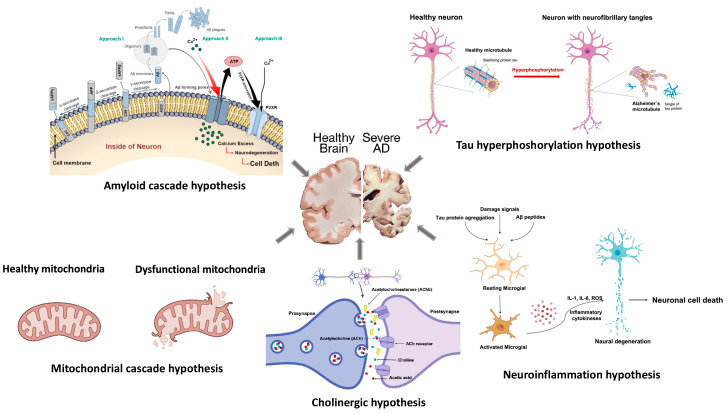
Different hypotheses of Alzheimer’s disease.

The current brief review summarizes the major hallmarks of Alzheimer’s disease (see Figure 1), which include the amyloid cascade hypothesis, tau protein hyperphosphorylation, mitochondrial cascade hypothesis, cholinergic hypothesis, as well as neuroinflammation, that have been proposed to explain its etiology. Secondly, it discusses current and traditional diagnostic and therapeutic methods and the potential impacts of nanotechnology to enhance the early detection and treatment of AD. This is of great significance as monitoring and rapid intervention can significantly improve the outcomes and prognosis of patients affected by AD. Thirdly, this review explores the challenges that need to be overcome in the development and implementation of nanotechnology-based approaches for AD. Finally, the article offers valuable information and insights for researchers and healthcare professionals interested in the evolving areas of clinical neuroscience, AD, and related nanotechnology.

## 2. Amyloid Cascade Hypothesis

### 2.1. Description of the Amyloid Cascade Hypothesis and Its Implications for Alzheimer’s Disease

The most extensively studied hypothesis of the progression of AD is the amyloid cascade hypothesis [8,9]. It is strongly believed that the loss of the neurons and their connections is caused by the neurotoxic effect of the amyloid-β peptide (Aβ).

**Figure 2:**
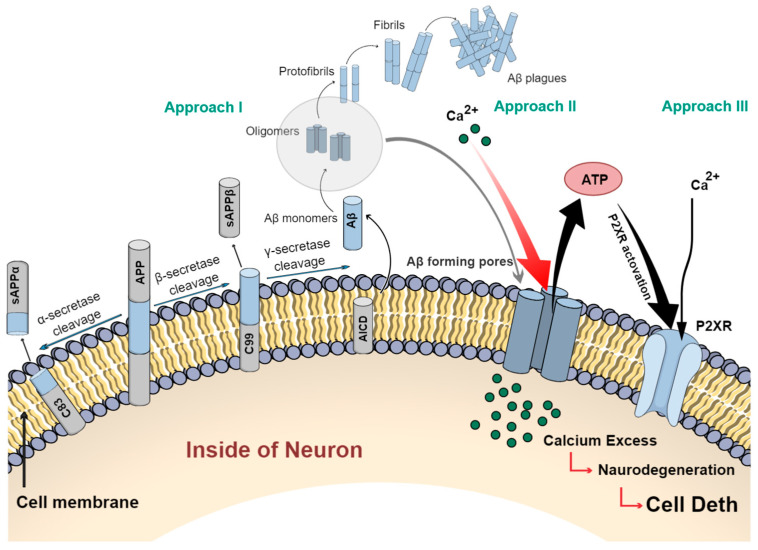
Schematic illustration of the concept of Aβ cytotoxic actions in neurons. The cleavage of APP protein and Aβ generation (on the left); Aβ aggregation (on the top); the creation of the calcium channels, release of ATP, and P2X receptor activation (on the right).

The accumulation of the peptide in the brain is the initial event of the process of dementia (see Figure 2 Approach I). Aβ peptides are generated by the metabolism of amyloid precursor protein (APP). APP is cleaved by special secretase enzymes (α-secretase, which liberates a soluble extracellular not amyloidogenic fragments, sAPPα, β-secretase, which releases mutant fragments, sAPPβ, and the protein fragment C99). C99 is subsequently cleaved by γ-secretase and releases the neurotoxic Aβ peptide. Aβ exists in various lengths, including the most abundant form consisting of 40 amino acids (Aβ40) and a less soluble 42 amino acid form (Aβ42). Both forms of Aβ40/42 have been found to kill neurons. Further, Aβ peptides aggregate to form oligomers, protofibrils, fibrils, and plaques. It is believed that this is the central event in the pathogenesis of Alzheimer’s, and additionally, the oligomeric form of Aβ has been suggested as the most toxic [10].

The other more consistent hypothesis is that when oligomers of Aβ peptides interact with the cell membrane, they form an ion channel-like structure similar to a pore (see Figure 2 Approach II). These channels accelerate ionic permeability, particularly for calcium, which further increase the intracellular Ca^2+^ level via the constant influx through the plasma membrane [11,12,13]. Years of research have supported the concept that the dysregulation of intracellular calcium homeostasis or calcium overload can trigger many downstream neurotoxic cascades, which leads to the increased production of toxin-reactive oxygen species, mitochondrial dysfunction, and the activation of genetic signals that affect synaptic stability and functions, leading to rapid neurodegradation and eventually cell death. Therefore, it has been suggested that, actually, the Aβ channels/pores inducing the entry of Ca2+ into cells may be responsible for the neurotoxic properties of Aβ and play a key pathological role in the neurodegenerative disorder associated with AD [14,15].

The third approach, directly related with the concept of the Ca^2+^-conducting Aβ channels, is the hypothesis of the uncontrolled release of ATP (adenosine triphosphate) from the cells (see Figure 2 Approach III) [16]. ATP is an important energetic molecule, an extracellular ligand of autocrine signaling, intercellular communication, and neurotransmission, with numerous physiological and pathophysiological roles. Normally, large and negatively charged ATP molecules cannot simply diffuse across the lipid bilayer of the plasma membrane. The conductive ATP release from the cytosol into the extracellular space is only possible through ATP-permeable channels. This homeostasis can be deregulated by the presence of Aβ channels. It has been demonstrated that those channels can work as non-selective pores with high permeability for metabolites such as ATP [17,18]. They increase the transport of ATP through the plasma membrane and enhance its extracellular concentration, inducing the activation of the ionotropic P2X receptors (especially its subtype P2X2, highly permeable to Ca^2+^) and trigger their further overexpression [10,11,18]. Therefore, the activation of P2XRs by ATP leakage could act as a secondary source of Ca^2+^ entry, accelerating the process of continued Ca^2+^ chain reactions and progressive damage of the neural network.

### 2.2. Discussion of Current Diagnostic and Therapeutic Approaches Related to the Amyloid Cascade Hypothesis

The majority of diagnostic tools related to the amyloid cascade hypothesis that are currently used include different techniques to evaluate the presence and the aggregation of Aβ in the brain [19].

- One of the commonly applied techniques to monitor Aβ is positron emission tomography (PET), which uses specific radiotracers that bind to the peptide [20,21,22]. It allows us to visualize the brain and see the accumulation of Aβ. Examples of well-known radiotracers for PET imaging are Pittsburgh Compound B (PiB), florbetapir, flutemetamol, and florbetaben, which binds specifically to Aβ [23,24]. Aβ PET imaging is not only used for diagnostic purposes but can also help in clinical trials for evaluating the efficacy of potential treatments for AD. The radiotracers can help identify patients who are appropriate for clinical trials of drugs targeting Aβ, such as amyloid-lowering agents.
- Another diagnostic tool that helps to evaluate Aβ levels in the brain is cerebrospinal fluid (CSF) analysis [25,26]. CSF is obtained through a lumbar puncture, and Aβ levels can be measured using enzyme-linked immunosorbent assays (ELISAs). Low concentrations of Aβ in the CSF have been shown to be associated with an increased risk of AD. This test is invasive but can provide valuable diagnostic information.
- Magnetic resonance imaging (MRI) is a non-invasive imaging technique that uses a magnetic field and radio waves to produce detailed images of the brain [27,28,29]. While MRI cannot directly measure Aβ, it can be used to detect changes in brain structure and function that may be associated with AD, including the shrinkage of the hippocampus, which is an area of the brain important for memory. Additionally, changes in brain activity in response to different stimuli might be monitored using functional MRI (fMRI) [30]. Studies have suggested that Aβ accumulation in the brain can affect brain activity and connectivity, which may be detectable using MRI and fMRI.
- Electroencephalography (EEG) is another non-invasive test that measures the electrical activity of the brain [31]. Some studies have suggested that Aβ accumulation in the brain can lead to changes in brain wave patterns that can be detected using EEG [32,33].
- Researchers are currently investigating whether blood tests could be truly valid to detect Aβ levels in the brain [34,35]. One approach involves measuring concentrations of Aβ in blood plasma, while another approach involves measuring the amount of Aβ oligomers, which are thought to be particularly toxic to neurons. This method could potentially provide a less invasive and more affordable diagnostic tool than, for example, PET imaging.
- In the last few years, it has been investigated whether Aβ accumulation in the retina of the eye or tears could be used as a biomarker for AD [36,37,38,39]. It has been shown that retinal imaging performed via optical coherence tomography (OCT) and fluorescence lifetime imaging ophthalmoscopy (FLIO) can indeed be used to detect changes in the retina that may be associated with AD.
- One important diagnostic method is also gene testing. Certain genetic mutations are known to increase the risk of developing AD, including mutations in the genes that encode Aβ and the presenilin proteins [40,41]. Gene testing can be used to identify individuals who carry these mutations and may be at increased risk of developing AD [42].
- Another tool used during each diagnostic analysis is neuropsychological test which helps to evaluate general cognitive function in patients with AD [43]. This test may include assessments of memory, language, attention, and executive function. However, while it does not directly measure Aβ, it can provide important information about the severity and progression of the disease.
- In addition to neuropsychological testing, cognitive assessments such as the Mini-Mental State Examination (MMSE) or the Montreal Cognitive Assessment (MoCA) can be used to assess cognitive function and screen for dementia [44,45].
- Finally, because no single diagnostic method for AD is completely accurate, researchers are exploring the use of multimodal biomarker approaches that combine several diagnostic evaluations. For example, combining PET imaging of Aβ with CSF analysis and neuropsychological testing may provide a more precise examination of AD [46,47]. Additionally, artificial intelligence (AI) algorithms are being developed to analyze data from multiple diagnostic tools to improve their efficiency. For example, machine learning algorithms can be trained to analyze brain imaging data, such as PET or MRI, to detect patterns of Aβ accumulation that may be associated with the disease [48,49].

Overall, it is important to note that while these diagnostic examinations are useful tools for assessing cognitive function and detecting changes in the brain associated with Aβ, none of them alone can definitively predict the disease. A comprehensive diagnostic evaluation may include a combination of several of these tests, along with a thorough medical history, physical examination, and laboratory testing.

Regarding current therapeutic approaches related to the amyloid cascade hypothesis, they include the following:

- Immunotherapy, which involves the use of antibodies to target and reduce the amount of Aβ peptide or its aggregation in the brain. Several monoclonal antibodies, such as aducanumab or lecanemab, have been developed and evaluated in clinical trials [50,51].
- Another approach consists of small molecule inhibitors, drugs that aim to prevent the formation of Aβ peptides or reduce their accumulation in the brain [52,53]. Examples include beta-secretase enzyme BACE inhibitors, which target the enzyme involved in the production of Aβ [54,55].
- An important and modern therapeutic tool includes anti-amyloid vaccines that can stimulate the immune system to produce antibodies that target and clear Aβ peptides from the brain [55,56].
- Gene therapies are focused on modifying the expression of genes involved in the production or clearance of Aβ peptides [57,58]. One approach involves using adeno-associated virus (AAV) vectors to deliver therapeutic genes directly to the brain [59,60].
- Potential for reducing the accumulation of Aβ and improving brain cognitive function is also shown by nutritional interventions and a complementary daily diet with certain nutrients, such as omega-3 fatty acids, antioxidants, and B vitamins. Nutritional interventions can work through various mechanisms, such as reducing inflammation and oxidative stress in the brain [61,62].
- A less common, and one of the most invasive, approaches involves stem cell therapies. They intend to replace damaged or dysfunctional cells in the brain with healthy cells. Researchers are exploring the use of stem cell therapies to replace or repair brain cells affected by AD, including those involved in the production and clearance of Aβ peptides [63,64].
- Lifestyle modifications, such as regular exercise, a healthy diet, and cognitive stimulation, have been also shown to reduce the risk of AD and may help to prevent or slow down the accumulation of Aβ protein in the brain.
- Approaches which are not exactly specified for Aβ peptides but are very important in the field of treatment of AD are brain stimulation techniques, such as transcranial magnetic stimulation (TMS) and transcranial direct current stimulation (tDCS). They are being explored as potential treatments for AD [65,66]. These techniques aim at improving cognitive function through activating different areas of the brain, including those involved in learning and memory.
- Similarly, specific drugs (cognitive enhancers) aim at improving cognitive function in AD patients through targeting neurotransmitter systems involved in learning and memory, such as acetylcholine and glutamate [67]. While not directly targeting Aβ, cognitive enhancers may help to mitigate the decline associated with the accumulation of peptides in the brain.

Many researchers believe that, similar to diagnostics methods, combination therapies target different aspects of the disease and may be more effective than a single approach. For example, combining immunotherapy with small molecule inhibitors or lifestyle modifications may have a synergistic effect. While the amyloid cascade hypothesis has been the focus of much AD research, it is still a topic of debate and controversy in the field. It is important to note that while many of these therapeutic approaches have shown promise in preclinical and clinical studies, there is still much research to be carried out to determine their safety and efficacy in treating AD.

### 2.3. Overview of Nanotechnology-Based Approaches Related to the Amyloid Cascade Hypothesis

Nanotechnology-based approaches have been explored to target different stages of the amyloid cascade hypothesis and may hold promise for the treatment of AD. A majority of them focus on the development of multifunctional nanoparticles with various purposes, such as targeting Aβ, reducing its production, and promoting neural regeneration. The most studied ones include the following:

- Nanoparticles for inhibiting Aβ aggregation [68], i.e., nanoparticles engineered to prevent Aβ aggregation and reduce its toxicity. Examples might include gold nanoparticles functionalized with polyphenols or graphene quantum dots [69,70].
- Nanoparticle-based delivery of Aβ antibodies [71], i.e., nanoparticles designed to deliver Aβ antibodies to the brain, where they can bind to and neutralize Aβ aggregates. This approach has shown promise in preclinical and clinical studies [72].
- Nanoparticle-based delivery of Aβ oligomer inhibitors [73,74,75]. Nanoparticles can help to deliver inhibitors of Aβ oligomerization to the brain, where they can prevent the formation of toxic Aβ species.
- Nanoparticle-based delivery of metal chelators. Metals such as copper and iron have been shown to play a role in Aβ aggregation and neurotoxicity [76,77]. Nanoparticles may transport metal chelators to the brain, where they can sequester these metals and prevent their interaction with Aβ.
- Nanoparticle-based delivery of small molecule drugs [78,79]. Specific drugs that target different stages of the amyloid cascade have been developed, but their efficacy is limited by poor brain penetration and off-target effects. Nanoparticles can be produced to improve brain targeting by these drugs, bypass BBB, and minimize their side effects. Researchers have developed nanoparticles as nanocarriers that can specifically bind to Aβ plaques and deliver drugs to clear them. For example, liposomes can be used to encapsulate drugs, deliver them to specific regions of the brain, and break down Aβ [80].
- Nanoparticle-based immunotherapy [81,82]. Nanoparticles are also engineered to stimulate the immune system to clear Aβ from the brain. They mimic the structure of Aβ, triggering an immune response to clear the plaques.
- Nanoparticle-based imaging agents, i.e., nanoparticles which serve as contrast agents in imaging techniques such as MRI or computed tomography (CT) scans [83]. They can detect Aβ deposits in the brain, making them visible in MRI or CT scans [84]. This can enable the early detection and monitoring of AD and facilitate the development of further therapies. Additionally, nanoparticles help to visualize peptides in the brain using imaging probes such as quantum dots that can selectively bind to Aβ peptides and allow their detection and visualization in vivo [70].
- Nanoparticle-based gene therapy for promoting Aβ clearance [58,85,86]. Nanoparticles can be designed to encapsulate therapeutic genes and deliver them directly to the brain. Once in the brain, the nanoparticles release the genes, which can then be taken up by brain cells. They promote the expression of proteins that are involved in the clearance of Aβ peptides.
- Nanosensors for early detection and monitoring. Another nanotechnology-based approach involves sensors for the early detection and monitoring of Aβ peptides in the brain and body fluids [87,88,89,90]. Nanosensors allow for the detection of Aβ at very small concentrations and for the recognition of specific proteins or various biomarkers that are associated with the formation of Aβ plaques. One approach to develop nanosensors for AD involves the use of nanomaterials such as graphene oxide, carbon nanotubes, or gold nanoparticles. These materials can be functionalized with specific antibodies or aptamers that can selectively bind to Aβ, allowing for its detection and quantification in biological samples. Another approach involves the use of nanoscale transistors and other electronic devices that can detect changes in electrical conductivity or other physical properties in response to binding to Aβ. These devices can be integrated into microfluidic systems. Nanotechnology-based sensors also offer the potential for real-time monitoring, for example, through using nanoscale probes that can detect changes in fluorescence or other optical properties in response to Aβ peptide aggregation.
- Nanoscale ultrasound. Nanoscale ultrasound is an example of an efficient method for breaking up Aβ aggregates [91,92]. The ultrasound-mediated elimination of Aβ plaques uses low-frequency ultrasound to induce vibrations in the brain tissue, disrupting the structure of Aβ aggregates and facilitating their clearance by the body’s immune system.
- DNA origami. Another option is DNA origami for creating nanoscale scaffolds and Aβ clearance [93,94,95]. DNA origami can be designed to produce nanoscale structures that can mimic the natural clearance mechanisms in the brain, such as the action of enzymes that break down Aβ peptides. Through attaching enzymes or other clearance-promoting molecules to the DNA origami scaffold, it may be possible to enhance the clearance of Aβ peptides from the brain.
- Stem cell therapy. Stem cell therapy apply nanotechnology to enhance the survival and integration of transplanted stem cells in the brain [96]. It involves the delivery and targeting of stem cells to specific areas of the brain affected by AD. For example, stem cells can be combined with nanoscale technologies, loaded on or encapsulated within nanoparticles, which can then be engineered to target specific cell types or regions of the brain. One potential approach is to use stem cells to produce and release factors that can promote the clearance of Aβ peptides from the brain. For instance, stem cells can be genetically engineered to produce enzymes that can break down Aβ peptides or cytokines that can stimulate immune cells to clear Aβ peptides from the brain. Another approach is to use stem cells to regenerate damaged or lost neurons in the brain. Stem cells can differentiate into various types of brain cells, including neurons, and can potentially replace damaged or lost neurons in the brain.
- Combinational therapy. Additionally, nanotechnology-based approaches are being investigated in combination with other therapies, such as gene therapy and stem cell therapy, to enhance their effectiveness. For example, nanoparticles can be used to deliver gene therapy to the brain to promote the production of factors that can clear Aβ or promote neural regeneration.

These approaches demonstrate the broad range of nanotechnology-based strategies. They hold great promise for the development of therapies and diagnostic tools related to the amyloid cascade hypothesis. The field is rapidly evolving, and new approaches are constantly being developed and refined.

### 2.4. Summary of Key Findings on Nanotechnology-Based Approaches and Future Directions for Research

The amyloid cascade hypothesis suggests that AD is caused by the accumulation of Aβ peptides and subsequent formation of Aβ oligomers or plaques in the brain. Nanotechnology-based approaches have been currently explored as a potential solution for addressing this hypothesis, with the following key findings:

- Nanoparticles have been developed as contrast agents for imaging Aβ plaques in vivo, allowing for the earlier detection of AD.
- Nanosensors capable of detecting Aβ peptides in biological fluids and brain tissue allow for the real-time monitoring of their aggregation.
- Nanocarriers such as liposomes, dendrimers, and solid lipid nanoparticles have been investigated as delivery systems for Aβ-targeting drugs, including antibodies and small molecules.
- Nanotechnology-based approaches have been used to engineer molecules that can inhibit the aggregation of Aβ peptides, potentially slowing or preventing the formation of Aβ plaques.
- Metal nanoparticles have been explored as a potential therapeutic strategy to promote the clearance of Aβ plaques from the brain.
- The use of nanotechnology-based approaches for combination therapies targeting both Aβ and tau protein pathology in AD is an active area of research.
- Graphene oxide nanoparticles can be used to inhibit Aβ aggregation and reduce Aβ-induced cytotoxicity.
- It has been demonstrated that multifunctional nanocarriers play a potential role in simultaneously targeting Aβ plaques, inhibiting Aβ aggregation, and delivering anti-inflammatory drugs.

In summary, nanotechnology-based approaches hold great promise for addressing the amyloid cascade hypothesis, with potential applications in early detection, imaging, drug delivery, and therapeutic interventions. However, to fully understand and optimize these approaches in the context of AD, further research is needed. For example, there is a need to develop more effective and targeted nanocarriers for drug delivery across the BBB and into the brain. This would enable the more efficient and precise delivery of therapeutic agents to target Aβ oligomers and plaques and other pathological features of AD. Researchers could investigate the efficacy and safety of different types of nanoparticles, such as liposomes, dendrimers, or polymeric nanoparticles, for this purpose. Another approach is to investigate further nanosensors that can detect early-stage Aβ aggregates in the CSF, blood, or urine. For instance, explore the use of various nanomaterials, such as graphene or carbon nanotubes, as sensing elements, and optimize their sensitivity and specificity. Future research should also explore nanomaterials for disrupting Aβ aggregation. It is important to design nanoparticles that can interfere with Aβ agglomeration and prevent the formation of toxic oligomers and fibrils. Also here, researchers could investigate the use of different types of nanoparticles, such as metal nanoparticles or silica nanoparticles, and assess their effectiveness in disrupting Aβ accumulation and toxicity. Additionally, future directions for research might include deeper investigation of the use of nanotechnology-based approaches for immunotherapy, such as the development of vaccines or engineered nanoparticles that can stimulate the immune system to clear Aβ plaques and other pathological proteins in the brain. Also, exploring the targeted delivery of stem cells or other regenerative therapies to damaged regions of the brain in AD can significantly improve the treatment of the disease. Despite development, further research is needed in the area of monitoring the progression and treatment response in AD, using different imaging techniques to track the accumulation and clearance of Aβ plaques. Exploring the potential of nanotechnology-based approaches for combination therapies, for example, targeting both Aβ and tau protein pathology in AD, is another area of research that calls for further investigation. Improvement can also be implemented in the development of more biocompatible and safe nanomaterials for use as AD therapeutics. This might include reduced potential toxicity and immune response, crucial parameters for successful clinical translation. It could be a milestone in the application of many nanomaterials. Translating promising preclinical findings into critical trials and evaluating the safety and efficacy of nanotechnology-based approaches for AD diagnosis and treatment in human patients is a critical step in advancing our understanding and treatment of AD. Overall, there is a significant opportunity for nanotechnology-based approaches to improve the diagnosis and treatment of AD, and further research in this field is likely to yield exciting discoveries and innovations in the years to come.

## 3. Tau Protein Hyperphosphorylation

### 3.1. Description of the Tau Protein Hyperphosphorylation Hypothesis and Its Implications for Alzheimer’s Disease

The tau protein hyperphosphorylation hypothesis is the second of the major theories which explain the development and progression of AD. It suggests that an abnormal process of hyperphosphorylation of the tau protein leads to the formation of large aggregates, neurofibrillary tangles (NFT), in the brain [6,97]. They disrupt normal cellular function, causing neuronal degeneration and eventual apoptosis [98,99].

**Figure 3:**
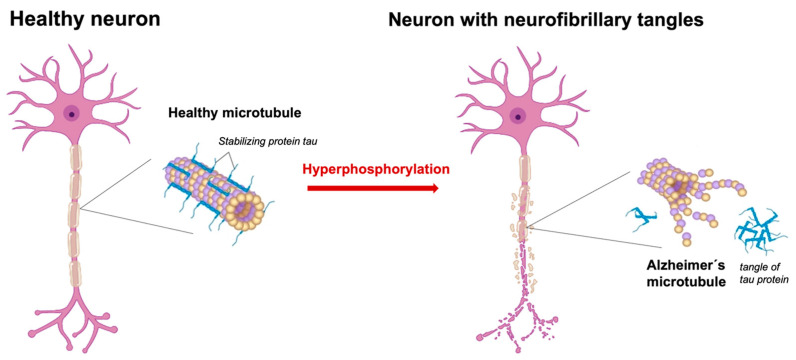
Pathophysiology of tau in Alzheimer’s disease. A healthy neuron and axonal microtubule stabilized by tau, which maintains the shape of axon (on the left). A neuron with tau aggregation, neurofibrillary tangles, and microtubule depolymerization (on the right).

The tau protein is a key component of the cytoskeleton of neurons, particularly microtubules, which provides structural support and facilitates the transport of nutrients and other substances from the cell to the axon and dendrites. In its normal state, the tau protein is regulated by various enzymes, including kinases and phosphatases, which add or remove phosphate groups from the protein [98]. Kinases such as glycogen synthase kinase 3β (GSK-3β) and cyclin-dependent kinase 5 (CDK5) are involved in tau phosphorylation, while phosphatases, such as protein phosphatase 2A (PP2A), are involved in tau dephosphorylation. In AD, the tau protein undergoes hyperphosphorylation, leading to the accumulation of abnormally phosphorylated tau in the form of paired helical filaments (PHFs), which ultimately form NFTs (see Figure 3).

In this state, tau proteins are losing their capacity to maintain normal microtubule morphologies and functions. Further, NFTs induce harmful reactive oxygen species (ROS) production and cellular degeneration. This extensive damage to the microtubule structure affects neurons and impairs normal axonal transport, resulting in poor signaling and brain lesions.

Several studies have provided evidence that the NFTs affect neuronal function and cause cognitive decline. One of the most reliable, the post-mortem examination of AD brains has confirmed that NFTs are closely associated with regions of the brain involved in memory and cognitive processes, and the degree of their accumulation correlates with the severity of the impairment [100]. Additionally, mutations in the tau gene have been associated with an increased risk of developing AD, providing further evidence for the role of tau in this pathogenesis [101].

However, while the tau protein hyperphosphorylation hypothesis is well established, and targeting tau over-phosphorylation has emerged as a promising therapeutic strategy for AD, the relationship between tau pathology and other hallmarks of AD, such as Aβ accumulation and mitochondrial dysfunction, is still not fully understood. Recent studies have suggested that Aβ accumulation can induce tau hyperphosphorylation and aggregation, contributing to the spread of tau pathology in the brain. Furthermore, evidence suggests that mitochondrial dysfunction can also exacerbate tau pathology, leading to neurodegeneration.

### 3.2. Discussion of Current Diagnostic and Therapeutic Approaches Related to the Tau Protein Hyperphosphorylation Hypothesis

In recent years, there has been significant progress in developing diagnostic approaches related to the tau protein hypothesis, specifically to detect tau pathology and assess its severity in patients.

- In some of studies, it has been found that a combination of tau and Aβ biomarkers can improve the accuracy of AD diagnosis, even in its early stages [102].
- Furthermore, advances in PET imaging using radiotracers that bind to tau protein have allowed for the visualization of its aggregates in vivo, providing valuable insights into disease progression and enabling earlier diagnosis [103,104,105]. A recent study also demonstrated the utility of tau–PET imaging in tracking the spread of tau pathology in the brain and predicting cognitive decline in AD patients [106,107].
- CSF biomarkers, such as tau and phosphorylated tau, have also been used as promising diagnostic tools [108,109]. CSF tau levels have shown to correlate with the degree of the pathology in the brain and can help differentiate tauopathies from non-tauopathies [110,111]. However, despite the high sensitivity of these measurements, CSF collection is invasive and not well-tolerated by some patients, and the interpretation of the protein concentrations is affected by various factors such as age, sex, and comorbidities.
- On the other hand, blood-based biomarkers for tau pathology have been explored as a less invasive and more convenient diagnostic approach [112,113]. Recent studies have shown that plasma tau levels are elevated in patients with AD and can help distinguish between AD and non-AD dementia [114,115,116]. For instance, researchers reported that higher levels of plasma tau were associated with greater cognitive decline and increased risk of mild cognitive impairment or dementia [115]. Additionally, it was found that plasma tau biomarkers had high accuracy in predicting Aβ positivity and discriminating individuals with AD from those without AD [116]. These findings suggest that plasma tau levels may be a useful biomarker for the early diagnosis and differentiation of AD. However, the accuracy and reliability of blood-based tau biomarkers are still being evaluated, and further validation is needed before their widespread use in clinical practice.
- In addition to these diagnostic approaches, efforts are also underway to develop novel biomarkers and imaging techniques to improve the early diagnosis and monitoring of tau hyperphosphorylation. For example, researchers are exploring the use of retinal imaging and other non-invasive biomarkers to detect early signs of tau pathology in AD [117,118].

In conclusion, few diagnostic approaches are available nowadays for the detection and monitoring of tau pathology in patients with AD. PET imaging with tau-specific radiotracers, CSF biomarkers, and blood-based biomarkers has shown promise in the diagnosis and differentiation of tau pathology. However, further validation and development of these approaches are needed to improve their accuracy and reliability for clinical use.

Regarding the tau hypothesis and current therapeutic approaches, there are several that have been developed, including the following:

- Instant tau immunotherapy is a method that aims at targeting and eliminating pathological forms of tau protein using antibodies. Several clinical trials are underway for tau immunotherapy, including passive immunotherapy, active immunotherapy, and immune checkpoint inhibitors [119,120,121,122].
- Another commonly used approach is the application of tau aggregation inhibitors. They are small molecules that target tau accumulation and prevent the formation of toxic aggregates. Few compounds have been identified that can inhibit tau aggregation, and preclinical studies have shown promising results [123,124]. Clinical trials of tau-targeted therapies are currently ongoing, with several promising candidates in the pipeline [125,126,127,128].
- Another potential solution also involves neuroprotective agents, or compounds that intend to protect neurons from tau-mediated toxicity. Several analytes have been identified that can protect against tau toxicity, including antioxidants, anti-inflammatory agents, and compounds that enhance autophagy [129].
- A promising technique, still in development, is gene therapy. The technique focuses on modifying the expression of genes involved in the tau pathway to reduce the formation of pathological tau protein. Several gene therapies are in preclinical development, targeting genes such as tau, glycogen synthase kinase 3 beta (GSK-3β), and microRNAs [101].
- Researchers also provide evidence for the high molecular diversity of tau contributing to the clinical heterogeneity of AD, which highlights the importance of personalized treatment approaches [130].

In conclusion, the tau protein hypothesis has generated considerable interest in the development of new therapeutic approaches for neurodegenerative diseases. The majority is still in the preclinical or early clinical stages.

### 3.3. Overview of Nanotechnology-Based Approaches Related to the Tau Protein Hyperphosphorylation Hypothesis

This area of intense research involves the use of nanotechnology to address the accumulation of tau protein in the brain [131]. The most promising approaches include the following:

- The nanoparticle-based delivery of tau-targeting therapeutics [78,132], or nanoparticles that can be specially designed to encapsulate and transport drugs that target tau pathology. For example, nanoparticles have been developed to deliver small molecule inhibitors of tau aggregation, such as methylene blue and curcumin, to the brain [133,134,135,136]. This approach could provide more targeted and effective delivery, minimizing off-target effects and increasing drug efficacy [137,138,139,140,141].
- Immunotherapy is another method that has shown promise in targeting tau pathology. Nanoparticles can be engineered to deliver antibodies that specifically target tau protein aggregates and stimulate an immune response against them. This could potentially provide a more targeted and effective way to deliver antibodies to the brain. Additionally, researchers highlight the advantages of immunotherapy over traditional drug therapies, including a better ability to target specific protein aggregates and elicit a sustained immune response, providing longer-term therapeutic effects [142,143].
- Nanoparticle-based imaging agents, or nanoparticles that can be used to detect and visualize tau pathology in the brain. Researchers are exploring the use of various types of nanoparticles, including quantum dots, gold, and iron oxide nanoparticles, for this purpose. [144,145,146] They can be designed to specifically bind to tau protein aggregates, allowing for their detection using imaging techniques such as MRI and PET scans. This approach could potentially aid in the early detection and diagnosis of AD, allowing for earlier intervention and treatment [147,148,149].
- Nanotechnology-based biosensors are another promising approach for the detection and monitoring of tau protein in biological fluids such as CSF or blood [150,151,152]. These biosensors can be designed to detect specific tau protein isoforms, including phosphorylated tau, which are known to be associated with AD. There are two main types of nanotechnology-based biosensors that are being investigated for tau detection: optical biosensors and electrochemical biosensors. Optical biosensors rely on the detection of light signals to measure changes in the target molecule, while electrochemical biosensors detect changes in electrical current or potential. Optical biosensors typically use fluorescent or luminescent nanoparticles, such as quantum dots or gold nanoparticles, as the detection platform. These nanoparticles are functionalized with tau-specific antibodies or aptamers, which bind to the target tau protein in biological fluids. When they bind, it causes a change in the fluorescence or luminescence of the nanoparticle, which can be measured and quantified.

Electrochemical biosensors, on the other hand, typically use a working electrode functionalized with tau-specific antibodies or aptamers. When the target tau protein binds to the electrode, it causes a change in the electrical current or potential, which again can be measured and quantified. Both optical and electrochemical biosensors have the potential to be highly sensitive and specific for the detection of tau protein in biological fluids. They could be used as diagnostic tools for AD, as well as for monitoring disease progression and treatment response.

- Gene-based therapies. Additionally, several gene-based therapies have been proposed for the treatment of tau pathology in AD, including gene therapy, RNA interference (RNAi), and CRISPR-Cas gene editing [153,154,155]. These therapies aim at targeting the underlying genetic mechanisms involved in the abnormal accumulation and hyperphosphorylation of tau protein. Gene therapy involves the delivery of specific genes that can regulate the expression of tau and other proteins involved in AD pathology. RNAi is another approach that uses small RNA molecules to selectively silence the expression of specific genes, including tau. CRISPR-Cas gene editing is a more recent development that involves manipulating the genome to correct genetic mutations or remove disease-causing genes.

Overall, among different nano-based approaches, the use of nanoparticles as imaging agents and drug delivery vehicles holds great potential for the development of effective diagnosis and targeted therapies for AD. The ability to detect tau pathology at an early stage and deliver therapeutics directly to the affected areas of the brain could significantly improve the treatment and management of this debilitating disease. However, further research is needed to optimize the design and delivery of nanoparticles, as well as to determine their safety and efficacy in clinical trials.

### 3.4. Summary of Key Findings on Nanotechnology-Based Approaches and Future Directions for Research

In conclusion, the tau protein hyperphosphorylation hypothesis continues to be an important and active area of research in AD. There are ongoing efforts to develop new biomarkers enhancing rapid diagnosis and effective treatment and which significantly impact the ability to prevent or slow down the progression of the disease. One promising avenue of research is nanotechnology-based approaches, which offer several potential benefits over traditional strategies, including targeted and more efficient drug delivery, as well as the early detection and monitoring of AD. Some of the major findings are listed below:

- The potential of using nanoparticles to deliver drugs and other therapeutic agents directly to the brain. Through encapsulating drugs for tau pathology, they can be protected from degradation and cleared more slowly from the body, allowing for sustained release and longer-lasting effects.
- Nanoparticles can also be used as imaging agents to detect and monitor the accumulation of tau protein in the brain.
- Nanoparticles can be designed to bind specifically to tau protein aggregates and other biomarkers of AD, allowing for the earlier detection and monitoring of disease progression.
- Research has also demonstrated the feasibility of using nanotechnology-based techniques for gene therapy and other emerging technologies, such as CRISPR-Cas gene editing, to target the underlying genetic causes of AD. Through delivering genes or RNA molecules that regulate the expression of specific proteins, including tau, or using CRISPR-Cas gene editing to modify or delete disease-causing genes, these approaches could potentially provide a more targeted and personalized treatment for AD.
- Nanoparticles can also be designed to address the heterogeneity and complexity of AD pathology through targeting multiple molecular mechanisms and pathways simultaneously. For example, nanoparticles can be designed to simultaneously target tau protein aggregates and Aβ peptides.
- In addition, the possibility of using nanotechnology-based methods to deliver multiple therapeutic agents simultaneously has also been shown. Through encapsulating different drugs or therapeutic agents in the same nanoparticle, they can be delivered simultaneously to the brain and work synergistically to slow disease progression.

Overall, these key findings suggest that nanotechnology-based approaches have the potential to impact the diagnosis and treatment of tau pathology in AD. However, there are still many challenges and limitations that must be addressed, and further research is needed to optimize these approaches for clinical use. Future research directions should focus on addressing them, including the development of stable and biocompatible nanoparticles, long-term safety and efficacy studies, and integration with existing therapies. Additionally, further research is needed to fully understand the underlying causes and mechanisms of tau and AD pathology and the potential role of nanotechnology-based approaches in targeting these driving processes. Future investigations should also explore the potential of combining nanotechnology-based approaches with other emerging technologies, such as machine learning and AI, to further enhance the precision and efficacy of treatment delivery [156,157]. AI can be used to analyze large datasets and identify patterns and biomarkers of the disease. Additionally, AI can be used to develop personalized treatment plans based on individual patient characteristics, such as genetic risk factors and the stage of the illness. Another area of research that requires attention is the development of non-invasive imaging techniques to monitor the distribution and accumulation of nanoparticles in the brain. This will enable researchers to better understand the pharmacokinetics and pharmacodynamics of nanotechnology-based approaches and optimize their design for maximum therapeutic benefit. Finally, research should also focus on addressing the potential social and ethical implications of nano-based strategies for AD. These include concerns regarding the affordability and accessibility of these treatments, as well as issues related to privacy, consent, and patient autonomy. Therefore, a multidisciplinary approach involving scientists, healthcare providers, policymakers, and ethicists is essential to ensure that nanotechnology-based approaches are developed and implemented in a responsible and equitable manner. In addition, future research should also focus on developing more sensitive and specific diagnostic tools for the early detection and monitoring of tau pathology. Nanoparticle-based biosensors and imaging agents could offer several advantages over existing methods, such as improved sensitivity and specificity, lower cost, and higher throughput. These tools could enable the earlier detection and diagnosis of AD, allowing for earlier intervention and potentially better treatment outcomes.

## 4. Mitochondrial Cascade Hypothesis

### 4.1. Description of the Mitochondrial Cascade Hypothesis and Its Implications for Alzheimer’s Disease

**Figure 4:**
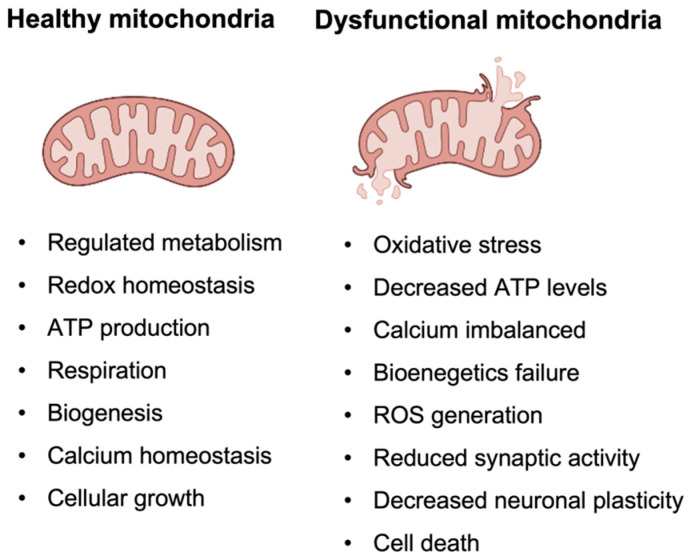
Potential causes and consequences of mitochondrial dysfunction, contributing to the development and progression of neurodegeneration.

The mitochondrial cascade hypothesis proposes that mitochondrial dysfunction is one of the key factors in the development and progression of AD [158]. Mitochondria are organelles responsible for energy production and maintenance of cellular homeostasis. The dysregulation of these processes can lead to a range of cellular dysfunctions, including increased production of ROS or affected calcium homeostasis. Eventually, they can contribute to neuronal damage and even cell death (see Figure 4) [159].

According to this hypothesis, on one hand, mitochondrial–bioenergetic dysfunction represents a byproduct of more fundamental AD events, such as chronic oxidative stress and the accumulation of Aβ peptides in the brain [158]. It is suggested that these processes lead to damage to mitochondrial membranes and impaired mitochondrial function, which in turn contribute to neuronal death and the development of AD pathology [158]. Oxidative stress results in the production of ROS that can damage mitochondrial proteins, lipids, and DNA, leading to organelle degeneration. Additionally, Aβ peptides can generate more ROS and therefore greatly contribute to the process. Moreover, Aβs bind to mitochondrial proteins, leading to impaired electron transport chain function and altered mitochondrial morphology and distribution. Aβ peptides can also cause mitochondrial permeability transition pore (mPTP) opening, which can result in the release of pro-apoptotic factors, such as cytochrome c, and ultimately lead to cell death [160]. Studies have reported that mitochondria isolated from the brains of AD patients have an increased content of Aβ peptides compared to those from healthy individuals. This might suggest that mitochondrial dysfunction induces the production of Aβ, which leads to increased ROS and a “vicious circle” of the cascade hypothesis [161].

On the other hand, researchers also argue that mitochondrial dysfunction could represent an independent or perhaps even primary event in AD [158]. One theory is that the process is caused by mutations in mitochondrial DNA (mtDNA). Several studies have reported that mtDNA mutations accumulate with age and are more frequent in individuals with AD. They can impair mitochondrial function, increase oxidative stress, and lead to neuronal death. Moreover, mutations in mitochondrial proteins have been linked to an increased risk of AD, further supporting the role of mitochondria in AD pathogenesis. Another theory suggests that impaired mitochondrial dynamics, such as mitochondrial fission and fusion, play a critical role in AD. Mitochondrial fission is the process through which a single mitochondrion divides into two or more smaller mitochondria, while fusion is the reverse process. Correct mitochondrial dynamics are essential for maintaining mitochondrial function and neuronal health. Disruptions of those processes have been observed in AD brains, and studies have suggested that the abnormal accumulation of Aβ peptides in the brain could additionally impair mitochondrial dynamics, leading to mitochondrial dysfunction and neuronal death. Recent studies have also highlighted the role of metabolic disabilities in AD, specifically the impairment of glucose metabolism. Glucose is the primary energy source for the brain, and studies have shown that its uptake and metabolism are impaired in individuals with AD, leading to reduced ATP production and increased oxidative stress [161].

### 4.2. Discussion of Current Diagnostic and Therapeutic Approaches Related to the Mitochondrial Cascade Hypothesis

Diagnostic approaches related to the mitochondrial cascade hypothesis are mainly focused on identifying markers of mitochondrial dysfunction that can be used to diagnose and monitor AD.

- One of the most promising biomarkers is oxidative stress, which is a result of the imbalance between the production of ROS and the cell’s ability to detoxify them [162]. Increased oxidative stress has been observed in both human AD brains and animal models of the disease, supporting the role of mitochondrial dysfunction in AD pathology. Biomarkers of oxidative stress that have been proposed include lipid peroxidation and protein carbonylation [163,164].
- Another potential indication of mitochondrial dysfunction is mtDNA damage. MtDNA is a small circular DNA molecule that is present in mitochondria and encodes several genes that are critical for mitochondrial function. Because mitochondrial dysfunction is a key feature of AD, it is thought that mtDNA mutations and damage may contribute to the development and progression of the disease. Some studies have reported that mtDNA damage is higher in AD patients, and that mutations may be associated with an increased risk of developing the disease. However, further research is needed to fully understand the potential of mtDNA as a diagnostic biomarker for AD [165].
- An approach also being investigated is the use of exosomes as biomarkers for AD [166,167]. Exosomes are small vesicles that are released by cells and contain a variety of proteins, lipids, and nucleic acids. Because exosomes can be isolated from biological fluids such as blood and CSF, they may provide a less invasive and more accessible diagnostic tool for AD. Some studies have reported that exosomes derived from AD patients contain markers of mitochondrial dysfunction, such as increased levels of oxidative stress and altered expression of mitochondrial proteins [168].
- In addition, imaging techniques including PET and MRI can provide important information on mitochondrial activity, such as the rate of glucose metabolism, one of the indicators of mitochondrial function [163].

Overall, while the mitochondrial cascade hypothesis is still an area of active research and debate, it has provided important insights into the potential role of mitochondrial dysfunction in AD pathogenesis and has spurred the development of new therapeutic strategies targeting their activity.

Several of the most common therapeutic approaches related to the mitochondrial cascade hypothesis for AD are presented below:

- Mitochondrial-targeted therapies aim at improving mitochondrial function and reducing accumulation of ROS through targeting specific mitochondrial components, such as the electron transport chain or mPTP. Several compounds that specifically target mitochondria have been investigated as potential treatments for AD [169,170]. These include mitochondria-targeted antioxidants, such as MitoQ, coenzyme Q10, alpha-lipoic acid, and vitamin E, and mitochondria-targeted SS peptides [171,172].
- The application of compounds that stimulate the production of new mitochondria, such as resveratrol, metformin and nicotinamide riboside, helps to compensate for the dysfunction of existing mitochondria. These activators may help improve mitochondrial function, increasing mitochondrial biogenesis or enhancing the production of ATP, and reduce the accumulation of damaged mitochondria [173].
- Mitochondrial fission/fusion modulators. The balance between mitochondrial fission and fusion is critical for maintaining mitochondrial health. This process can affect mitochondrial morphology and function [174]. Compounds that modulate these processes include mitochondrial division inhibitor 1 (Mdivi-1), which inhibits mitochondrial fission, and mitofusins (Mfn1 and Mfn2), which promote mitochondrial fusion [175,176].
- Anti-inflammatory therapies aim to reduce inflammation and oxidative stress in the brain through targeting immune cells and inflammatory pathways [177].
- Exercise and lifestyle interventions that promote regular exercise and healthy lifestyle habits such as a balanced diet, good sleep, and stress reduction have been shown to improve mitochondrial function and reduce the risk of AD [178].
- Another therapeutic approach related to the mitochondrial cascade hypothesis for AD is the use of mitophagy modulators. Mitophagy is a process through which damaged mitochondria are removed and recycled, promoting mitochondrial quality control. Dysregulation of mitophagy has been implicated in the pathogenesis of AD, and compounds that modulate this process may have therapeutic potential. For example, rapamycin and trehalose have been shown to induce mitophagy and reduce the accumulation of damaged mitochondria in AD models [179].
- A possible therapeutic approach might also be the use of stem cell therapies to replace damaged or dysfunctional neurons and restore mitochondrial function. Recent studies have shown promising results using induced pluripotent stem cells (iPSCs) to generate new neurons and improve cognitive function in AD models [180].

Overall, the mitochondrial cascade hypothesis for AD offers multiple avenues for therapeutic intervention. While more research is needed to fully understand the underlying mechanisms and determine the most effective treatments, targeting mitochondrial dysfunction and oxidative stress represents a promising approach for the development of new AD therapies.

### 4.3. Overview of Nanotechnology-Based Approaches Related to the Mitochondrial Cascade Hypothesis

Nanotechnology-based approaches have shown promise in the development of diagnostic and therapeutic interventions related to the mitochondrial cascade hypothesis for AD. These approaches involve the use of nanoparticles and other nanoscale materials to deliver drugs or therapeutic agents specifically to the mitochondria, allowing for the targeted treatment of mitochondrial dysfunction.

- Mitochondria-targeted antioxidants. This approach involves the use of mitochondria-targeted antioxidants, such as coenzyme Q10, encapsulated in nanoparticles for targeted delivery to the mitochondria. These nanoparticles can be designed to specifically target damaged mitochondria in the brain, reducing oxidative stress and promoting mitochondrial function [181].
- Conjugated nanoparticles. Nanoparticles can be designed to overcome the BBB through exploiting various mechanisms, such as receptor-mediated transcytosis or adsorptive-mediated transcytosis, which allow nanoparticles to cross the BBB and reach the brain [182]. One example of this approach is the use of liposomes to deliver mitochondria-targeted compounds to the brain [183]. Liposomes can be functionalized with various targeting ligands, such as transferrin or apolipoprotein E, which can enhance their uptake by brain cells and improve their efficacy in treating mitochondrial dysfunction in AD. Another example is the use of gold nanoparticles, which can be coated with a layer of polyethylene glycol (PEG) to improve their biocompatibility and stability in the body. Gold nanoparticles can be conjugated with various mitochondrial-targeting molecules, such as tri-phenyl-phosphonium (TPP) or ubiquinone, to enhance their uptake by mitochondria and improve their therapeutic efficacy.
- Gene therapy. Another approach is the use of nanoparticles to deliver small interfering RNA (siRNA) or antisense oligonucleotides (ASOs) to target genes involved in mitochondrial dysfunction [184]. For example, siRNA targeting amyloid precursor protein (APP) has been delivered using nanoparticles to reduce APP expression and improve mitochondrial function in AD models [185].
- Nanoparticles in drug delivery. Nanoparticles have also been used to deliver drugs targeting the mPTP, a key regulator of mitochondrial function and cell death. For example, a mitochondria-targeted derivative of cyclosporin A (CsA), a known inhibitor of the mPTP, has been encapsulated in nanoparticles and shown to improve mitochondrial function and reduce cell death [186].
- Targeting mitochondrial membrane potential. Nanostructures can be designed to target the mitochondrial membrane potential, which is a key regulator of mitochondrial function and has been found to be altered in AD. For example, cationic nanoparticles have been used to deliver peptides that target the mitochondrial membrane potential, leading to improved mitochondrial function and reduced oxidative stress [187].
- Imaging and diagnosis. In addition to drug delivery, nanotechnology-based approaches can also be used for imaging and diagnosis of mitochondrial dysfunction in AD. For example, quantum dots have been used to track mitochondrial dynamics and function. Another example is magnetic nanoparticles for MRI of mitochondrial function in the brain. Through targeting these nanoparticles specifically to the mitochondria, MRI can provide a non-invasive method for detecting and monitoring mitochondrial dysfunction in AD [188].
- Nanosensors. Another approach is the use of nanosensors to detect and monitor mitochondrial dysfunction in AD. Nanosensors are highly sensitive and selective devices that can detect small changes in molecular signals, such as ROS or mitochondrial membrane potential, two important indicators of mitochondrial dysfunction. Nanosensors can be designed to target specific subcellular compartments, such as mitochondria, and provide real-time monitoring of its activity.

Overall, nanotechnology-based approaches offer a promising avenue for the development of targeted therapies for AD related to mitochondrial dysfunction. These approaches can enhance the delivery of mitochondria-targeted compounds to the brain, improve their uptake by mitochondria, and provide real-time monitoring of mitochondrial function in live cells or animal models.

### 4.4. Summary of Key Findings on Nanotechnology-Based Approaches and Future Directions for Research

The mitochondrial cascade hypothesis proposes that mitochondrial dysfunction is one of the central features of AD pathogenesis. In recent years, nanotechnology-based approaches have emerged as a promising tool for the development of new diagnostic methods and therapies. Here are some key findings in this area:

- Nanoparticle-mediated delivery of therapeutic agents. Nanoparticles have been used to deliver therapeutic agents to the mitochondria, allowing for the targeted treatment of mitochondrial dysfunction.
- Mitochondria-targeting nanoparticles. Nanoparticles can be designed to specifically target the mitochondria, allowing for the precise delivery of therapeutic agents to these organelles. These nanoparticles can be engineered to selectively accumulate in the mitochondria due to their unique properties, such as size, surface charge, and surface chemistry.
- Nanosensors for monitoring mitochondrial function. Nanosensors can be used to monitor mitochondrial function in real time. For example, fluorescent nanosensors have been developed to monitor mitochondrial pH changes in response to oxidative stress.
- Nanoparticles for imaging mitochondria. Nanoparticles can also be used for imaging mitochondria in living cells. For example, quantum dot nanoparticles have been used to visualize the morphology and dynamics of mitochondria in real time.

Overall, further research is essential to gain a deeper understanding of the mechanisms underlying mitochondrial dysfunction in AD, especially the potential roles of Aβ, tau, and other factors. This might help researchers to gain insights into the pathogenesis of AD and develop novel strategies for treatment and prevention of the disease, for example, the production of more sensitive and specific nanoparticles for the detection of Aβ peptides and tau protein in the brain or the enhancement of non-invasive techniques, such as imaging, new biomarkers, and metabolic assays, to offer rapid, precise, and early indication of the disease. Future research might also focus on optimizing the delivery of drugs to the brain using nanoparticles, particularly those targeting mitochondrial dysfunction. The BBB still presents a significant challenge for drug delivery, but nanoparticles can be engineered to bypass this barrier and target specific cells and their components in the brain. This would allow for the targeted and effective therapy of AD, potentially slowing or even reversing disease progression. It is also important to investigate various interventions, including lifestyle changes, pharmacological agents, and gene therapies, that target the mitochondrial cascade in order to slow down or halt the progression of AD. Moreover, the heterogeneity of AD suggests that personalized approaches to treatment may be necessary. Mitochondrial dysfunction may manifest differently in different patients, and identifying their subgroups with specific symptoms can be essential.

## 5. Cholinergic Hypothesis

### 5.1. Description of the Cholinergic Hypothesis and Its Implications for Alzheimer’s Disease

Another important hypothesis in the development of AD is the cholinergic hypothesis [189]. It suggests that the loss of cholinergic neurons, which produce acetylcholine (ACh) in the brain, induces presynaptic cholinergic deficit and might be a cause of AD. ACh is a neurotransmitter involved in a wide range of cognitive processes, including attention, learning, and memory. Therefore, according to the hypothesis, decreased levels of ACh contribute to the impairment of all these functions [189].

**Figure 5:**
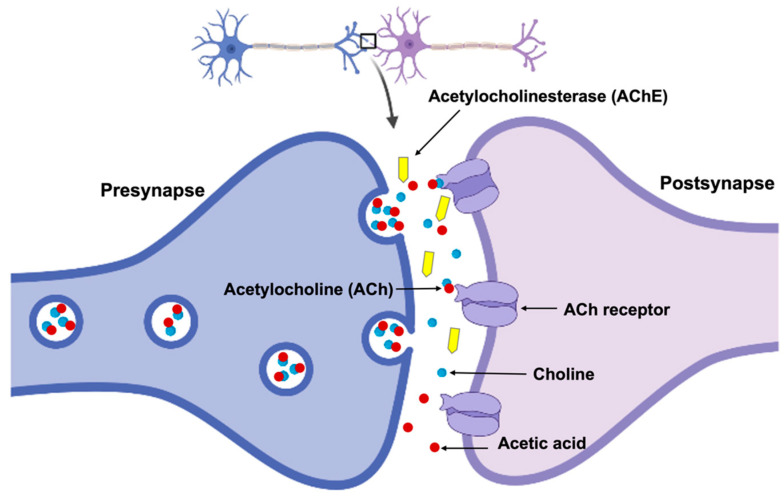
Neurotransmission of the cholinergic synapse. In the synaptic cleft of a cholinergic neuron, acetylcholine (ACh) is decomposed into choline and acetic acid by acetylcholinesterase (AChE).

Apart from the theory, the cholinergic hypothesis has been supported by a broad range of physical examinations, providing strong evidence for the importance of cholinergic neurotransmission in cognitive abilities and AD. For example, post-mortem investigations of people with AD have shown that there is a reduction in the number of cholinergic neurons and a decrease in the activity of cholinergic enzymes in the brain. Additionally, drugs that target the cholinergic system, such as cholinesterase (AChE) inhibitors, are commonly used to improve cholinergic transmission and treat AD. They are known for blocking the degeneration of Ach by AchE and have proved to make a positive impact on the cognitive functions of patients. An example of reduced neurotransmission of the cholinergic synapse when ACh is decomposed by AChE into choline and acetic acid is presented in Figure 5. Another example that proves the importance of cholinergic neurotransmission includes neuroimaging studies. They have demonstrated that AD patients have reduced cholinergic activity in certain brain regions, which is correlated with cognitive impairment. The theory has been also confirmed through genetic studies which have found that mutations of genes affecting cholinergic neurotransmission are associated with an increased risk of developing AD.

Similarly, studies of CSF in AD patients have found decreased levels of ACh and other cholinergic markers, suggesting a role for cholinergic dysfunction in the disease. Additionally, behavioral and cognitive tests in both animals and humans have shown that cholinergic drugs can increase activation in brain regions involved in cognitive processing. In summary, the cholinergic hypothesis has been supported by a wide range of evidence from various sources, including post-mortem studies, animal studies, neuroimaging studies, genetic studies, and clinical trials of cholinergic drugs. These findings have led to the development of cholinesterase inhibitors as a first-line treatment for AD, which provides symptomatic relief through increasing the availability of ACh in the brain. However, while they proved to be effective in improving cognitive function in some people with AD, they do not work for everyone, and their effects tend to be modest. This suggests that the cholinergic hypothesis may not fully explain the complexity of AD. It is likely that multiple factors, including Aβ aggregation, tau protein tangles, and inflammation, contribute to the development and progression of the disease.

### 5.2. Discussion of Current Diagnostic and Therapeutic Approaches Related to the Cholinergic Hypothesis

Currently, there are several diagnostic approaches related to the cholinergic hypothesis:

- For instance, neuropsychological testing is often used to diagnose AD and examine cognitive impairment. Tests that specifically assess cholinergic function, such as the evaluation of attention and memory, may be helpful to support the hypothesis that AD is related to a deficiency in cholinergic neurotransmission [190].
- Another useful diagnostic tool is PET imaging. It measures the levels of acetylcholine in the brain [191]. One commonly applied PET tracer for this purpose is [11C]methyl-4-piperidinyl propionate ([11C]PMP), which binds to the enzyme responsible for acetylcholine synthesis. PET imaging is also commonly used to estimate the density of cholinergic receptors in the brain.
- Additionally, the concentration of acetylcholine in the brain can be analyzed based on the signal from CSF biomarkers [192]. For example, the CSF levels of acetylcholinesterase are decreased in patients with AD.
- Apart from CSF, blood biomarkers related to cholinergic dysfunction, such as acetyltransferase and acetylcholinesterase, have been identified. To control their different amounts is crucial for diagnosing and monitoring of AD.
- Neuroimaging techniques, such as MRI, can be used to detect brain atrophy in AD. Brain atrophy in certain regions, such as the hippocampus, is believed to be associated with cholinergic dysfunction and cognitive decline [193].
- Functional MRI is another neuroimaging technique that measures changes in the brain blood flow in response to different tasks or stimuli. fMRI can be used to analyze brain activity related to cholinergic processes such as cognitive functions [194].
- Electroencephalography (EEG) and magnetoencephalography (MEG) are non-invasive techniques used to measure the electrical activity in the brain [195]. These techniques can be used to assess brain function related to cholinergic activity, such as attention and memory processing.
- Another non-invasive technique is transcranial magnetic stimulation (TMS). This technique uses magnetic fields to stimulate specific areas of the brain [196]. TMS can be used to assess the brain to investigate its cholinergic activity and has also been shown to improve cognitive processes in AD patients.
- Additionally, genetic testing has recently been used to identify mutations in genes related to AD, such as the presenilin-1 and presenilin-2 genes [197]. These genes play a role in the production of Aβ peptides, which accumulate in the brains of Alzheimer’s patients and are thought to contribute to cholinergic dysfunction.
- Eye tracking can be used to assess visual attention [198]. Studies have shown that patients with AD have impaired eye movements during visual tasks, which may be related to cholinergic dysfunction.
- Different from the other methods, post-mortem examination of the brain can provide definitive confirmation of AD and its association with cholinergic dysfunction [199]. Histological examination of brain tissue can reveal the presence of Aβ plaques and neurofibrillary tangles, as well as changes in cholinergic markers, such as choline acetyltransferase and acetylcholinesterase.

In conclusion, while the cholinergic hypothesis provides a useful framework for understanding the underlying mechanisms of AD, analyzing this condition remains complex and multifaceted. A comprehensive diagnostic approach may involve a combination of clinical, cognitive, imaging, and biochemical assessments, tailored to the individual patient’s needs and symptoms.

The therapeutic approaches related to the cholinergic hypothesis are as follows:

- The common method currently applied involves cholinesterase inhibitors [200]. These drugs work through preventing the breakdown of acetylcholine, thereby increasing its availability in the brain. Some examples of cholinesterase inhibitors frequently used for the treatment of AD include donepezil, galantamine, and rivastigmine.
- Another approach proposes nicotinic receptor agonists [201]. Nicotine is a natural agonist of nicotinic acetylcholine receptors. Drugs that activate these receptors are being investigated as potential treatments for AD. For example, the drug varenicline, which is used to aid smoking cessation, is being studied for its potential cognitive-enhancing effects in the disease.
- One therapy for AD also considers using different ACh precursors such as choline [202]. They are compounds that the body can use to make acetylcholine and can be found in foods such as eggs and liver.
- Some researchers are investigating the use of stem cells to restore cholinergic function in the brain [203]. For example, neural stem cells can be engineered to produce acetylcholine and are then transplanted into the brain to restore cholinergic function.
- Another potential therapeutic strategy applies dual-acting compounds. These are drugs that combine cholinesterase inhibition with other mechanisms of action that may also be beneficial in AD [204]. For example, the drug memantine is an N-methyl-D-aspartate (NMDA) receptor antagonist that is often used in combination with cholinesterase inhibitors for the treatment of moderate to severe AD.
- In addition to drugs, there are also non-pharmacological interventions that may be beneficial in AD. For example, cognitive stimulation programs that involve activities such as puzzles, games, and reminiscence therapy have been shown to improve cognitive function in people with the disease. It is thought that these types of interventions may enhance cholinergic function through promoting the release of acetylcholine in the brain.
- Researchers are also investigating the use of gene therapies to restore cholinergic function in the brain. For example, gene therapy approaches that involve the delivery of genes encoding cholinergic enzymes or receptors to the brain are being studied as potential treatments for AD.

Overall, while there are a range of current therapeutic approaches related to the cholinergic hypothesis, it is important to note that AD is a complex condition, and it is unlikely that any one approach will be sufficient to provide a cure. As such, research into this disease is ongoing, and new treatments are being developed and tested all the time.

### 5.3. Overview of Nanotechnology-Based Approaches Related to the Cholinergic Hypothesis

Nanotechnology-based approaches are constantly being developed to address the cholinergic hypothesis and to provide potential therapeutic solutions for AD. Some of them include the following:

- Targeted drug delivery entails the use of nanoparticles to deliver drugs directly to the brain [205]. For example, nanoparticles can be engineered to encapsulate drugs that enhance cholinergic neurotransmission, such as acetylcholinesterase inhibitors [206]. This can be achieved through engineering the surface of nanoparticles with ligands that can recognize and bind to cholinergic receptors. Once bound, nanoparticles can release drugs or other therapeutic agents that can modulate cholinergic neurotransmission [207]. These nanoparticles can be designed to target specific regions of the brain, such as the hippocampus, where cholinergic neurons are particularly affected in AD.
- Nanosensors. Another nanotechnology-based approach is to develop nanosensors that can detect cholinergic neurotransmission in real time [208]. For example, researchers are exploring the use of nanowire sensors that can measure the levels of acetylcholine in the brain. These sensors can be implanted providing continuous monitoring of cholinergic neurotransmission, which can help researchers better understand the dynamics of this neurotransmitter system in health and disease [209].
- Nanowire electrodes. The development of nanoscale devices that can selectively stimulate cholinergic neurons in vivo also has an important impact. For example, researchers have designed brain-implantable nanoelectronic devices (nanowire electrodes) that can simulate the activity of cholinergic neurons using light or electrical signals. These devices can be controlled externally and provide a targeted approach to enhance cholinergic neurotransmission in the brain. The approach has been shown to improve cognitive function in animal models of AD [210,211].
- Scaffolds. Nanotechnology also involves developing scaffolds for the tissue engineering of cholinergic neurons [212]. Tissue engineering is based on creating three-dimensional structures that can support the growth and function of cells or tissues. Researchers are developing nanoscale scaffolds that can mimic the structure and function of the extracellular matrix in the brain and promote the differentiation and growth of cholinergic neurons. These scaffolds can be used to replace or repair damaged or lost cholinergic neurons in AD.
- Biomarkers. The development of nanotechnology-based diagnostic tools that can detect biomarkers associated with cholinergic dysfunction has also been reported. For example, researchers have developed gold nanoparticles that can detect levels of acetylcholinesterase in blood samples, which may be a biomarker for AD. These diagnostic tools can be used to detect early signs of cholinergic dysfunction and monitor the progression of the disease.
- Non-invasive stimulation. Finally, this nano-based strategy is used to develop novel therapies that can modulate cholinergic neurotransmission in a targeted and controlled manner. For example, researchers are exploring the use of nanoscale magnetic particles that can selectively activate cholinergic neurons in the brain using magnetic fields [213]. This approach can provide a non-invasive and targeted way to enhance cholinergic neurotransmission in AD.

Overall, nanotechnology-based approaches related to the cholinergic hypothesis offer exciting opportunities to develop new therapies, diagnostic tools, and delivery systems for AD.

### 5.4. Summary of Key Findings on Nanotechnology-Based Approaches and Future Directions for Research

The cholinergic hypothesis proposes that the degeneration of cholinergic neurons in the brain is responsible for the cognitive impairment associated with AD. Here are some key findings on nano-based strategies related to this hypothesis:

- Nanoparticles can be designed to specifically target cholinergic neurons and to deliver cholinergic agents directly to the brain, enhancing their effectiveness and minimizing side effects.
- Nanoparticles can be used to deliver gene therapy for cholinergic neurons, which can promote their growth and survival or replace damaged neurons.
- Nanobased approaches show promise in improving cognitive function in animal models of AD through increasing acetylcholine levels in the brain.
- Various types of nanostructures, including liposomes, dendrimers, and nanogels, have been explored for their potential in cholinergic-based therapies for AD.
- Nano-based approaches can be used to overcome the limitations of traditional drug delivery methods, such as poor solubility, low bioavailability, and short half-life.
- Research is ongoing to explore more the potential of non-pharmacological interventions, such as transcranial magnetic stimulation and cognitive training, to enhance cholinergic function in AD.
- Other nano-based approaches include the use of nanosensors for detecting biomarkers of AD and the development of nanocarriers for gene therapy.

One potential future direction for addressing AD is to focus on developing new and more effective cholinergic therapies. This could involve exploring strategies to promote the regeneration of cholinergic neurons, which are known to be affected in patients with the disease. Additionally, a better understanding of the molecular mechanisms underlying cholinergic degeneration in AD is crucial, especially in improving diagnostic methods and treatment. Another important area of research is the identification of new biomarkers that can accurately reflect cholinergic degeneration and predict disease progression. This could lead to the earlier recognition of damaged parts of the brain and allow more rapid and effective interventions. It is also important to investigate the interaction between the cholinergic system and other neurobiological factors, such as inflammation and oxidative stress, in the development and progression of AD. This could provide insights into potential new treatment targets and approaches. Exploring the ability of non-pharmacological interventions, such as physical exercise and cognitive stimulation, to enhance cholinergic function in AD is another promising area of research. These interventions may have great impact on improving cognitive function and slowing disease progression, potentially complementing pharmacological therapies.

Overall, developing new cholinergic therapies, identifying new biomarkers, investigating the interaction between the cholinergic system and other neurobiological factors, and exploring non-pharmacological interventions are all important future directions for addressing AD.

## 6. Neuroinflammation Hypothesis

### 6.1. Description of the Neuroinflammation Hypothesis and Its Implications for Alzheimer’s Disease

**Figure 6:**
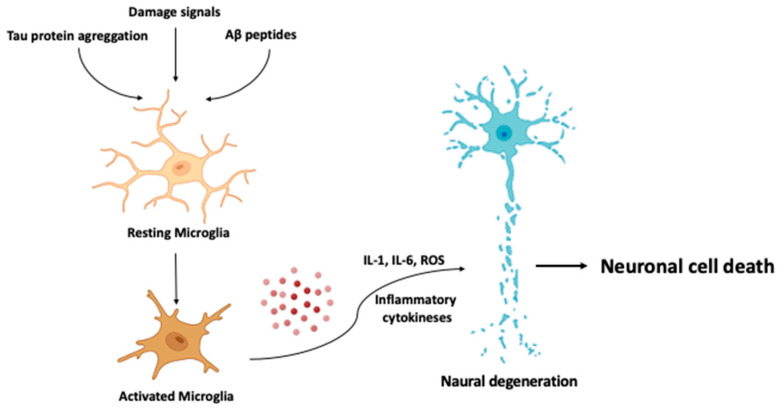
Microglia-mediated neuroinflammation in Alzheimer’s disease (AD). Under the pathology of AD, the accumulation of Aβ peptides and tau NFT induce microglial-like activation, which produces inflammatory cytokines, releases ROS, and causes neuronal cell death.

The neuroinflammation hypothesis of AD proposes that chronic activation of the brain’s immune system, specifically microglia, triggers the production of cytokines and other inflammatory mediators, and contributes to the development and progression of the disease [214,215]. A schematic illustration of microglia-mediated neuroinflammation in AD is presented in Figure 6. Microglia are the resident immune cells located throughout the central nerve system. They account for around 15% of the total brain cellular population. In healthy conditions, microglia are in an inactive, “resting” state or maintaining tissue homeostasis under physiological conditions, while inflammation results in their immediate activation. Reactive microglia changed their phenotype, release ROS, and produce inflammatory cytokines such as IL-1 and IL-6. They localize and migrate to the affected place to eliminate damaged cells.

It is hypothesized that one of the primary drivers of the activation of microglia is the presence of Aβ peptides and their abnormal accumulation. Activated microglia respond to Aβ, resulting in migration to aggregates and the phagocytosis of Aβ. A Number of investigations have demonstrated that activated microglia phagocytose Aβ; however, these microglia become enlarged and, after prolonged periods, are no longer able to process Aβ. Additionally, recent studies suggest that tau pathology can activate microglia in a similar manner to the presence of Aβ peptides. The accumulation of hyperphosphorylated tau protein within neurons triggers an immune response from microglia. This response can include their activation and the release of inflammatory cytokines. Furthermore, chronic activation of microglia can also lead to an excessive and sustained inflammatory response, which contributes to the progression of the disease.

In addition to microglial stimulation, tau pathology has also been linked to the activation of astrocytes, another type of immune cell in the brain. Astrocytes can also release inflammatory molecules, contributing to the neuroinflammatory response.

The implications of the neuroinflammation hypothesis for AD are significant. If chronic neuroinflammation is a key driver of the disease, then targeting the immune system may be a promising approach for treating or preventing the disease.

### 6.2. Discussion of Current Diagnostic and Therapeutic Approaches Related to the Neuroinflammatory Hypothesis

The neuroinflammation hypothesis has led to the development of several diagnostic approaches to detect neuroinflammation in the brain.

- One commonly used diagnostic approach is PET imaging, which uses radioactive tracers to detect the presence of neuroinflammation markers in the brain and to track the progression of neuroinflammation over time [216]. PET imaging can detect changes in the levels of various biomarkers such as translocator protein (TSPO) and glial fibrillary acidic protein (GFAP), which are indicators of the condition. It can also provide valuable information about the dynamics of neuroinflammation and how it is related to disease progression [217,218].
- Recent advances in molecular biology and genomics are providing new opportunities for identifying novel biomarkers of neuroinflammation. For example, researchers are exploring the use of epigenetic markers, microRNAs, and other non-coding RNAs as potential indicators of neuroinflammation [219,220]. They may be more sensitive and specific than traditional biomarkers and give new insights into the mechanisms of the disease.
- Another approach is CSF analysis, which can provide data on the levels of biomarkers such as cytokines, chemokines, and complement factors, which are indicative of neuroinflammation [109,221].
- In addition, MRI can be used to detect changes in the brain’s structure and function that are associated with neuroinflammation. For example, diffusion tensor imaging (DTI) can detect the changes in white matter integrity that are associated with the condition, and fMRI can be used to detect differences in brain activity during the process [222,223].
- Another approach for detecting neuroinflammation is the use of blood biomarkers, the substances that are released into the bloodstream in response to inflammation in the brain. They can be measured using a blood test, and their levels can indicate the presence and severity of the condition. Examples of blood biomarkers that are associated with neuroinflammation include C-reactive protein (CRP), interleukin-6 (IL-6), and tumor necrosis factor-alpha (TNF-α) [224,225].
- Furthermore, advances in AI and machine learning techniques are allowing researchers to analyze large datasets of imaging and biomarker data to identify patterns and associations that may be missed by traditional methods [226,227]. This approach, known as radiomics, has the potential to improve the accuracy of neuroinflammation diagnosis, prediction of disease progression, and treatment response.

Overall, the development of new diagnostic approaches related to the neuroinflammation hypothesis is important for improving the understanding of the role of inflammation in neurological disorders and for developing new treatments that target neuroinflammation.

Regarding the therapeutic approaches related to the neuroinflammation hypothesis, they can include several ones that are listed below:

- Nonsteroidal anti-inflammatory drugs (NSAIDs). NSAIDs are commonly used to reduce inflammation in the body and can also be effective in reducing inflammation in the brain [228,229]. However, the long-term use of NSAIDs may have side effects, such as gastrointestinal bleeding.
- Another approach might be the use of immunomodulatory drugs that target the immune system and can reduce inflammation in the brain. Examples include interferon-beta, glatiramer acetate, or natalizumab, which are also used to treat multiple sclerosis [230,231].
- Steroids such as prednisone are also important in the process [232]. However, the long-term use of steroids can also have side effects, such as weight gain and increased risk of infections.
- One drug-based therapeutic strategy involves modulating microglial activation or function [233]. Some drugs such as minocycline, which have shown to reduce microglial activation, are being studied for their potential therapeutic effects in neuroinflammatory diseases.
- Stem cell therapy has shown promising results in reducing inflammation in the brain and promoting neurodegeneration. Mesenchymal stem cells, in particular, have anti-inflammatory properties and have been studied for their potential therapeutic effects in neuroinflammatory diseases [234,235].
- Non-invasive brain stimulation techniques such as transcranial magnetic stimulation (TMS) and transcranial direct current stimulation (tDCS) have been shown to reduce inflammation in the brain and improve symptoms of the neuroinflammatory condition [236,237].
- Cannabinoids such as cannabidiol (CBD) and delta-9-tetrahydrocannabinol (THC) have anti-inflammatory properties and may have potential therapeutic effects in neuroinflammatory diseases [238,239].
- A healthy diet, rich in anti-inflammatory supplements, and lifestyle can help reduce inflammation in the body and the brain [240]. Applying a diet rich in fruits, vegetables, and omega-3 fatty acids and engaging in regular exercise can help to decrease risk of the condition.
- Mind–body therapies such as meditation and yoga have been shown to reduce inflammation in the body and may have similar effects in the brain [241]. These therapies can also help reduce stress, which can contribute to inflammation.
- Precision medicine involves tailoring treatment to an individual’s specific genetic, environmental, and lifestyle factors [242,243]. With advances in technology such as genomic sequencing, precision medicine may help identify the underlying causes of neuroinflammation and guide personalized treatment approaches.
- Given the complex nature of neuroinflammation and its potential involvement in various neurological and psychiatric disorders, combination therapies may be more effective than single therapies alone. For example, a combination of anti-inflammatory drugs, immunomodulatory drugs, and lifestyle changes may be more effective in reducing inflammation and improving symptoms than any single therapy alone.

It is important to note that the neuroinflammation hypothesis is still being studied, and more research is needed to determine the most effective therapeutic approaches. Additionally, some of these approaches may have potential side effects and may not be appropriate for everyone.

### 6.3. Overview of Nanotechnology-Based Approaches Related to the Neuroinflammatory Hypothesis

Nanotechnology-based approaches have shown promise in the diagnosis, monitoring, and treatment of neuroinflammatory conditions. The major strategies include the following:

- Nanosensors. One potential application of nanotechnology in this field is the development of nanosensors that can detect biomarkers of inflammation in the brain [244]. These sensors can be designed to detect specific molecules, such as cytokines, that are indicative of the condition. This technology could allow for the earlier identification of neuroinflammatory processes and more accurate monitoring of disease progression.
- Nano-based targeted drug delivery. Nanoparticles have also been investigated as potential drug delivery vehicles for the treatment of neuroinflammatory conditions. They can be designed to target specific cells in the brain, such as microglia, which are key players in the inflammatory response [245]. Through delivering anti-inflammatory drugs directly to these cells, it may be possible to reduce inflammation in the brain while minimizing side effects.
- Gene therapy. In addition to drug delivery, nanoparticles can also be used to deliver genetic material to cells in the brain [246]. This approach, known as gene therapy, has the potential to target specific genes involved in the inflammatory response and modulate their expression. This technology is still in the early stages of development, but it has the potential to revolutionize the treatment of neuroinflammatory conditions.
- Nanoparticle-based imaging agents. Another area where nanotechnology-based approaches have shown promise in the context of neuroinflammation is in the development of imaging agents [247]. Specifically, nanoparticles can be designed to carry imaging agents, such as fluorescent dyes or contrast agents for MRI, to the site of inflammation in the brain. This technology can be used to visualize the extent and location of neuroinflammation, which is crucial for accurate diagnosis and monitoring disease progression.
- Nanoparticles can also be designed to penetrate the BBB [248]. This is particularly important for the treatment of neuroinflammatory conditions, as many anti-inflammatory drugs are unable to penetrate the BBB [249,250]. Through designing nanoparticles that can cross the BBB, it may be possible to deliver drugs directly to the brain and target inflammation more effectively.
- Nanotechnology-based approaches can also be used to develop implantable devices that can monitor and modulate neuroinflammation in real-time [251]. For example, nanoparticles can be incorporated into implantable devices to detect changes in cytokine levels, which can then trigger the release of anti-inflammatory drugs. These devices have the potential to provide continuous, personalized treatment for neuroinflammatory conditions.
- Another area where nanotechnology-based approaches can be used in the context of neuroinflammation is in the development of vaccines [252]. Specifically, nanoparticles can be designed to carry antigens and adjuvants to stimulate an immune response against inflammatory factors.
- Furthermore, nanotechnology-based approaches can be used to develop strategies to reduce inflammation and promote tissue repair [253,254,255]. For instance, nanoparticles can be designed to mimic extracellular matrix components, such as laminin, which can support nerve cell growth and regeneration. Additionally, nanoparticles can be functionalized with anti-inflammatory agents, growth factors, or other bioactive molecules to promote tissue repair and regeneration in the brain.
- Finally, nanotechnology-based approaches can also be used to improve the understanding of the mechanisms underlying neuroinflammation. For example, nanoparticles can be used to label and track immune cells, such as microglia, in the brain. This technology can provide insights into how these cells interact with others in the brain and how the interaction contributes to neuroinflammation and disease progression.

In conclusion, nanotechnology-based approaches have the potential to provide significant advances in the diagnosis, monitoring, and treatment of neuroinflammatory conditions. While many of these technologies are still in the early stages of development, they offer promising avenues for improving our understanding of neuroinflammation and developing effective therapies for neurodegenerative diseases.

### 6.4. Summary of Key Findings on Nanotechnology-Based Approaches and Future Directions for Research

The neuroinflammation hypothesis suggests that chronic inflammation in the brain may significantly contribute to the development and progression of AD. Nanotechnology has emerged as a promising approach for developing new diagnostic tools and therapies in this field. Some key findings related to the use of nanotechnology in the context of neuroinflammation and AD include the following:

- Nanoparticles can be used to enhance the delivery of imaging agents to the brain, potentially improving the ability to detect and monitor neuroinflammatory changes in AD.
- Nano-based approaches can be used to develop gene therapies that target specific genes involved in the neuroinflammatory response in AD.
- Nanoparticles can be designed to specifically target activated microglia and astrocytes in the brain, which are key components of the neuroinflammatory response in AD. The targeted delivery of anti-inflammatory agents to these cells could potentially reduce neuroinflammation and improve cognitive function.
- Gold nanoparticles have been shown to have anti-inflammatory properties and could potentially be used as therapeutic agents to reduce neuroinflammation in AD.
- Iron oxide nanoparticles can be used as contrast agents in MRI imaging to detect neuroinflammatory changes in the brain.
- Nanoparticles can be used to deliver siRNA or other genetic material to brain cells, potentially allowing for the targeted knockdown of genes involved in the neuroinflammatory response in AD.
- Quantum dots can be used as fluorescent probes to detect and monitor the activity of microglia and astrocytes in the brain.
- Polymeric nanoparticles can be designed to encapsulate or conjugate with anti-inflammatory agents and target the brain to reduce neuroinflammation. The controlled targeted delivery of anti-inflammatory agents to the brain could potentially reduce systemic side effects and improve drug efficacy.
- Magnetic nanoparticles can be used to magnetically manipulate and sort activated microglia and astrocytes in the brain. This technology could potentially be used to isolate and study these cells, leading to a better understanding of the neuroinflammatory response in AD.
- Nanoparticles can be used to deliver growth factors or other neurotrophic agents to the brain, potentially promoting neuronal regeneration.

Overall, these findings highlight the potential of nanotechnology for developing new approaches to understand and target neuroinflammation in AD. However, more research is needed to fully explore the potential of these technologies and develop effective nano-based therapies for AD.

Future research in this area may involve further developing nanotechnology-based tools for monitoring and modulating neuroinflammation. Nanoparticles can be engineered to target specific cells or molecules involved in neuroinflammation and can be used as imaging agents to visualize particular conditions in the brain. Furthermore, optimized nanoparticles can be designed to deliver therapeutic agents specifically to sites of inflammation, potentially reducing the side effects associated with systemic drug administration. Additionally, it is crucial to further investigate the role of neuroinflammation in neurodegenerative diseases. There is growing evidence that neuroinflammation plays a key role in the development and progression of neurodegenerative diseases including AD. Further research could explore the mechanisms underlying this connection and investigate whether nanotechnology-based interventions could be used to modulate neuroinflammation and slow disease progression. Future research should also involve further development and optimization of drug delivery systems for treating neurological disorders. More nanoparticles can be designed to cross the BBB and target specific cells or molecules in the brain, providing the controlled release of analytes. While nanotechnology has enormous potential for treating neurological disorders, there are also concerns about the potential toxicity of nanomaterials in the brain. Further research is needed to fully understand the risks associated with nanotechnology-based interventions and to develop strategies for minimizing any potential harm.

## 7. Conclusions and Discussions of the Potential Benefits and Limitations of Nanotechnology-Based Approaches for the Diagnosis and Treatment of Alzheimer’s Disease

AD is a complex and multifactorial neurodegenerative disorder that still remains poorly understood. Over the years, several hypotheses have been proposed to explain the underlying mechanisms of the disease. While each hypothesis provides valuable insights into the knowledge on AD, none of them fully explain its hidden nature. It is likely that a combination of these hypotheses, along with other factors, will contribute to the development and progression of AD. Further research is needed to gain a better understanding of the disease’s underlying mechanisms and to develop effective treatments.

Herein, we presented an overview of different diagnostic and therapeutic strategies related to AD. It has been highlighted that among all approaches, nanotechnology-based approaches have the potential to revolutionize the diagnosis and treatment of AD through providing more accurate and effective methods for detecting and treating the underlying pathologies of the disease. To summarize, we would like to demonstrate some of the major potential benefits, limitations, and challenges for the diagnosis and treatment of AD. The benefits include the following:

- Early detection and diagnosis. One of the major advantages of nanotechnology-based approaches is their ability to detect Aβ peptides, hyperphosphorylated tau protein, cytokines associated with neuroinflammation, chemokines, and other biomarkers in the brain or body fluids such as CSF, blood, and urine. This can be performed even before clinical symptoms of the disease appear. Nanoparticles can be engineered to bind specifically to these biomarkers and generate signals that can be detected and quantified using imaging techniques such as MRI or PET. Early detection can provide an opportunity for rapid intervention and better therapy management.
- Targeted drug delivery involves nanoparticles that can be engineered (functionalized with peptides or antibodies) to target specific cells or regions of the brain affected by AD. They can carry drugs that can inhibit the production and accumulation of Aβ or hyperphosphorylated tau protein or promote their clearance. In the case of the neuroinflammation hypothesis, nanoparticles can be designed to target cells and tissues affected by inflammation, such as microglia and astrocytes. They can also enhance mitochondrial function or protect mitochondria from oxidative stress and dysfunction. For example, polyphenol-coated nanoparticles can scavenge free radicals and prevent mitochondrial damage in neurons. Moreover, nanoparticles loaded with mitochondria-targeting antioxidants may prevent their dysfunction and improve cognitive ability. Overall, targeted drug delivery can improve the efficacy of drug therapies while minimizing side effects.
- BBB penetration is an approach to use nanoparticles in order to overcome the BBB, a protective barrier that prevents the entry of many drugs and therapeutic agents into the brain. This can allow for the more effective delivery of drugs and minimally invasive treatment.
- Improved pharmacokinetics. This can be achieved through engineering nanocarriers to influence the pharmacokinetic properties of drugs, such as longer half-life, increased stability, and reduced clearance. This strategy can improve the efficacy and safety of AD therapies.
- Reduced toxicity. This approach uses biocompatible nanoparticles to reduce the toxicity of drugs, encapsulating and delivering them specifically to the affected areas of the brain and minimizing their dosages.
- Versatility. Apart from biocompatibility, nanoparticles can be produced with a wide range of sizes, shapes, and surface properties, making them versatile tools for developing new treatments for AD.
- Enhanced imaging. Nanoparticles are also used as contrast agents to enhance imaging techniques such as MRI, PET, and CT scans. These imaging techniques can provide more accurate and detailed information about, e.g., the extent of Aβ accumulation, visualizing inflammation and disease progression in the brain.
- Multi-modal imaging. Nanoparticles may potentially offer multi-modal imaging capabilities, allowing for the simultaneous detection of multiple biomarkers and disease features. This can improve the accuracy and reliability of AD diagnosis and monitoring.
- Monitoring treatment efficiency. Nanotechnology-based approaches can enable real-time monitoring of the treatment efficiency for AD through detecting changes in, e.g., hyperphosphorylated tau protein levels. This can help to optimize therapy and improve patient outcomes.
- Non-invasive treatment. Some nanotechnology-based strategies such as magnetic nanoparticles or ultrasound can be used to treat AD without invasive procedures. For example, magnetic nanoparticles can be used to induce hyperthermia in Aβ deposits, which can break down Aβ peptides and reduce their toxicity. Non-invasive therapies also include the delivery of nanoparticles via, e.g., internasal or oral administration, reducing the need for invasive treatment.
- Biomarker discovery. Nano-based methods can help identify new biomarkers that can be used to diagnose AD at an early stage. For example, nanoparticles can be used to capture and analyze peptides or other biomolecules in biological fluids such as blood or CSF.
- Disease prevention. Nanotechnology-based approaches can potentially be used to prevent AD through targeting the early stages of Aβ accumulation in the brain. For example, nanoparticles can be designed to capture and remove Aβ peptides from the brain before they form aggregates.
- Personalized medicine. Nanotechnology-based approaches can potentially provide personalized medicine through tailoring treatments to the specific needs of individual patients based on their unique genetic and molecular profiles. For example, nanoparticles can be designed to carry drugs that target specific genetic mutations or molecular pathways associated with the production of Aβ. They also can target specific forms of hyperphosphorylated tau protein that are present in some patients but not others.
- Combination therapy. Nanotechnology-based approaches can be used in combination with other therapies such as immunotherapy or gene therapy to provide a more comprehensive treatment for AD. For example, nanoparticles can be engineered to carry drugs that enhance those therapies or to deliver multiple drugs simultaneously. This can increase the effectiveness of treatments and reduce the overall dose of each drug.
- Remote monitoring. Nanotechnology-based approaches can potentially provide remote monitoring of AD progression and treatment response. For example, nanoparticles can be engineered to emit signals that can be detected using wearable devices, allowing for the real-time monitoring of Aβ clearance in the brain.
- Improved safety. Nanotechnology-based approaches can potentially improve the safety of AD treatments through minimizing off-target effects and reducing the toxicity of drugs. For example, nanoparticles can be designed to specifically target Aβ peptides, minimizing the risk of damage to healthy cells in the brain.
- Improved brain function. Nanoparticle-based therapies may have the potential to not only slow down the progression of AD but also improve brain function through restoring, e.g., the cholinergic system and enhancing memory and cognitive function.
- Cost-effective treatment. Nanotechnology-based approaches can potentially provide cost-effective treatments for AD through reducing the amount of drug required for effective treatment and minimizing the need for invasive procedures.
- Improved understanding. Nanotechnology-based approaches can be used to gain a deeper understanding of the mechanism of AD, leading to the development of new therapeutic targets and strategies.

Therefore, nanotechnology-based approaches have the potential to provide a wide range of benefits for the diagnosis and treatment of AD. However, despite the potential for earlier and more accurate diagnosis, targeted drug delivery, enhanced imaging, non-invasive treatment options, disease prevention, BBB penetration, or improved pharmacokinetics, there exist limitations and challenges to be addressed. They include the following:

- Safety concerns. One of the major challenges with using nanoparticles for treatment is ensuring their safety. Nanoparticles can interact with cells and tissues in unexpected ways and may cause toxicity or immune responses. It is important to thoroughly test the safety of nanoparticles before they can be used in humans.
- Manufacturing and quality control. Another challenge with nanotechnology-based approaches is manufacturing and quality control. Nanoparticles need to be manufactured to strict specifications, and quality control measures must be put in place to ensure consistency and safety. Scaling up the production of nanoparticles can also be difficult, and there may be batch-to-batch variability.
- Targeting specific cells and molecules. Another challenge is ensuring that nanoparticles can target specific cells affected by AD and molecules such as Aβ peptides. The BBB can make it difficult for nanoparticles to enter the brain, and targeting specific compounds can be challenging.
- Efficacy. Even if nanoparticles can be targeted to specific cells, it is not yet clear whether they will be effective in treating AD. More research is needed to determine the optimal properties and dosing of nanoparticles for the treatment.
- Regulatory approval. Any nanoparticles for AD treatment will need to go through regulatory approval before they can be used in humans. This process can be lengthy and expensive, and there is no guarantee that nanoparticles will be approved for clinical use.
- Cost. The cost of developing and producing nanoparticles may be high. This could limit access to treatment for some patients and may also limit the commercial viability of nanoparticle-based therapies.
- Clearance mechanisms. One potential limitation of using nanoparticles is that the clearance mechanisms for these particles in the brain are not yet fully understood. It is possible that nanoparticles may accumulate in the brain or other organs over time and cause toxicity or interfere with other body functions.
- Variability in patient response. Another limitation is that patient responses to nanoparticles may vary due to differences in genetics, disease progression, or other factors. This could make it difficult to predict how well nanoparticles will work in different patients.
- Limited treatment window. There may be a limited window of opportunity for using nanoparticles to treat AD, as the disease may progress to a point where it is no longer reversible or treatable. This means that nanoparticles may need to be used in combination with other treatments or in earlier stages of the disease to be effective.
- Long-term effects. It is not yet clear what the long-term effects of using nanoparticles may be. It is possible that they could cause unintended side effects or have other long-term effects that are not yet understood.
- Accurate detection. Another challenge of nanotechnology-based approaches is the possible limitation of specific biomarker detection, such as Aβ peptide or tau protein. These biomarkers may not be present in significant amounts until later stages of the disease and may not accurately reflect the extent of the neurons’ degradation.
- Technical challenges: The design and production of nanoparticles with specific properties, such as size, shape, and surface chemistry, can be technically challenging. This can limit the scalability of nanotechnology-based approaches for treating AD.
- Variable effectiveness: The effectiveness of nanotechnology-based approaches can vary depending on the type and severity of AD, as well as individual patient factors. This can limit their overall efficacy and utility for treating AD.
- Difficulty targeting specific cells. Even with targeted drug delivery, it can be challenging to ensure that nanoparticles are taken up by specific cells, such as activated microglia and astrocytes. This can limit the efficacy of treatment and lead to off-target effects.
- Ethical considerations. The use of nanotechnology-based approaches for treating AD raises ethical considerations, such as the potential for unintended consequences and the need to ensure equitable access to treatment.
- Patient acceptance. Some patients may be hesitant to undergo treatment with nanotechnology-based approaches due to concerns about safety, efficacy, or the use of unfamiliar technologies.
- Compatibility with existing treatments. Nanotechnology-based approaches may not be compatible with existing treatments for AD, which can limit their utility as a standalone treatment or in combination with other treatments.
- Lack of long-term data. The long-term safety and efficacy of nanotechnology-based approaches for treating AD are not yet fully understood, and more research is needed to determine their effectiveness over time.

In conclusion, while nanotechnology-based approaches offer many advantages over traditional strategies for diagnosing and treating AD, there are also several limitations and challenges that need to be addressed to optimize their safety and efficacy for clinical use. Continued research and development are needed to overcome these challenges to fully implement nanotechnology-based approaches in treating AD.
